# Circulation of Shiga Toxin-Producing *Escherichia coli* Phylogenetic Group B1 Strains Between Calve Stable Manure and Pasture Land With Grazing Heifers

**DOI:** 10.3389/fmicb.2020.01355

**Published:** 2020-06-30

**Authors:** Leonard S. van Overbeek, Jan H. Wichers, Aart van Amerongen, Herman J. W. van Roermund, Patricia van der Zouwen, Peter T. J. Willemsen

**Affiliations:** Wageningen University and Research (WUR), Wageningen Research (WR), Wageningen, Netherlands

**Keywords:** *Escherichia coli*, animal production systems, calves, human pathogen, pasture, Shiga toxin, stable manure, Shiga toxin-producing *Escherichia coli* (STEC)

## Abstract

*Escherichia coli* strains carrying Shiga toxins 1 and 2 (*stx*_1_ and *stx*_2_), intimin (*eae*), and hemolysin (*ehx*A) production genes were found in grass shoot, rhizosphere soil, and stable manure samples from a small-scale cattle farm located at the center of Netherlands, using cultivation-dependent and -independent microbiological detection techniques. Pasture land with grazing heifers in the first year of sampling in 2014 and without grazing cattle in 2015 was physically separated from the stable that housed rose calves during both years. Manure from the stable was applied to pasture via injection into soil once per year in early spring. Among a variety of 35 phylogenetic distinctly related *E. coli* strains, one large group consisting of 21 closely resembling *E. coli* O150:H2 (18), O98:H21 (2), and O84:H2 (1) strains, all belonging to phylogenetic group B1 and carrying all screened virulence traits, was found present on grass shoots (10), rhizosphere soil (3), and stable manure (8) in 2014, but not anymore in 2015 when grazing heifers were absent. Presence and absence of these strains, obtained via enrichments, were confirmed via molecular detection using PCR-NALFIA in all ecosystems in both years. We propose that this group of Shiga toxin-producing *E. coli* phylogenetic group B1 strains was originally introduced via stable manure injection into the pasture. Upon grazing, these potential pathogens proliferated in the intestinal track systems of the heifers resulting in defecation with higher loads of the STEC strain onto the grass cover. The STEC strain was further smeared over the field via the hooves of the heifers resulting in augmentation of the potential pathogen in the pasture in 2014, whereas in 2015, in the absence of heifers, no augmentation occurred and only a more diverse group of potentially mild virulent *E. coli* phylogenetic group A and B1 strains, indigenous to pasture plants, remained present. Via this model, it was postulated that human pathogens can circulate between plants and farm animals, using the plant as an alternative ecosystem. These data indicate that grazed pasture must be considered as a potential carrier of human pathogenic *E. coli* strains and possibly also of other pathogens.

## Introduction

Human pathogenic bacteria such as Shiga toxin-producing *Escherichia coli* (STEC) strains can circulate across the different ecosystems relevant in plant production (van Overbeek et al., 2014). From there, these pathogens can enter the human food production chain, either directly via colonization of freshly consumable plants (Franz et al., 2008; Deering et al., 2012; van der Linden et al., 2013) or indirectly via colonization of plants that are used as animal feed in livestock production (Dodd et al., 2003; Williams et al., 2007). It seems likely that these pathogens circulate between plant and animal ecosystems via grazing, intestinal track colonization, excretion, and plant colonization.

Human pathogens often are transmitted to plant production systems via animal excrements, and the transmission route via manure application to soil may be the best studied contamination route of these pathogens to crop plants (van Overbeek et al., 2014). However, excrements of wild animals and (migratory) birds also may be held responsible for contamination of crop plants, either via droppings in production fields or via contamination of open surface water bodies that are used for irrigation (Atwill et al., 2015). Plants must be considered as alternative environments for human pathogens that are used as vectors for digestive track colonization of animals and humans via ingestion of plant-derived feed and food (Teplitski and de Moreas, 2018). Considered from the perspective of the pathogen, persistence in alternative environments such as plants is advantageous for eventual later transmission to intestinal track systems of other warm-blooded animals (van Elsas et al., 2011; Teplitski and de Moreas, 2018).

Plant contamination with human pathogens has considerable implications on human health as was demonstrated at the outbreak of the food-borne disease in Hamburg and surrounding area in 2011. The outbreak was caused by the enterohemorrhagic *E. coli* O104:H4 strain carrying Shiga toxin2a (*stx*_2__a_) genes (Mellmann et al., 2011). Although, this strain may be considered as an exceptional agent in food-borne outbreaks, it became clear that this highly virulent strain emerged from a less virulent variant of *E. coli* O104:H4 that was involved in a previous outbreak (Mellmann et al., 2011; Ahmed et al., 2012). Horizontal transmission of virulence genes played an important role in the rapid evolution of the outbreak strain, and it may be questioned whether horizontal transmission of virulence genes takes place in plant production systems, especially in those that are practiced under intensive management systems (Guttierrez-Rodriguez and Adhikari, 2018). In plant production systems, microbial communities from different ecosystems, such as from soil, manure, water, and plants amalgamate, and interactions between these communities in typical microbial hotspots such as the rhizospheres of crop plants were proposed to result in higher gene exchanges between microbes originating from the different habitats in van Overbeek et al. (2014). The fact that different plasmid-borne extended beta-lactamase resistances (ESBL) were found in different enteric species from herbal plants grown in Southeast Asia (Veldman et al., 2014) can be an indication for the fact that horizontal gene transmission to *E. coli* does occur in the phytobiome.

Shiga toxin-producing *Escherichia coli* (STEC) and *Salmonella enterica* can colonize plants from emerging seeds (Liu et al., 2018). Later during plant growth, contamination with these pathogens can occur via agricultural handlings such as at irrigation of plants (van der Linden et al., 2013) and at manure application to soil (Franz et al., 2008). Internalization of plants has been described for both enteric pathogens (Ávila-Quezada et al., 2010; Deering et al., 2012; Scott et al., 2017). Many commonalities in gene distribution exist among endophytic enterobacterial strains highlighting the conservation of functions that overlap with enteric human pathogens (Lòpez-Fernàndez et al., 2015). The enterohemorrhagic *E. coli* O157:H7 strain was found to be present in the apoplast of edible plants, and binding to the plant surface via curli and biofilm formation appeared to play an important role in the initial steps of colonization of internal compartments of plants (Wright et al., 2016). From studies on plant-colonizing *Salmonella enterica* cells, it appeared that the mechanisms used for infection of plants and animals are conserved (Schikora et al., 2011; Wiedemann et al., 2015; de Moraes et al., 2017; Cox et al., 2018). Inside plants, the invading enteric pathogens must protect themselves from the plant immune system, and genes were shown to be differentially expressed in *E. coli* O157:H7 cells exposed to spinach and lettuce extracts, and genes typically involved in stress responses were higher expressed (Crozier et al., 2016). Residence of *E. coli* K12 cells in lettuce plants also resulted in higher resistance to oxidative stress, but also to increased chemo-attraction to lettuce leaf extracts indicating that these are important features for *E. coli* cells residing in plants (de los Angeles Dublan et al., 2014).

In livestock animals, the intestinal track system must be considered as an important hot spot for microbial colonization and horizontal gene transmission (van Overbeek et al., 2014). Many different *E. coli* serotypes were found in pigs, and after pig slaughtering, some serotypes persisted throughout the entire pork food production chain (Colello et al., 2016). Different *E. coli* serotypes and biotypes were also found present in cattle feces (Bettelheim et al., 2005), and especially, the diet that the animals received played an important role in a selection of different *E. coli* types, including the ones that pose serious threats to humans upon oral ingestion such as *E. coli* O157:H7 (Hovde et al., 1999; Bettelheim et al., 2005; Callaway et al., 2009; Cote et al., 2015; Berry et al., 2017). From livestock animals, gut microorganisms enter arable soils upon manure applications. Survival of *E. coli* was higher in cattle manure-amended soil than in cattle manure itself (Oliver et al., 2006), and manure-to-soil ratio, temperature, and competition with soil-indigenous microbes were found to be important factors influencing the survival time of *E. coli* O157:H7 in manure-amended soil (Jiang et al., 2002). Mutations in the stress response-controlling gene *rpo*S in *E. coli* O157:H7 appeared to be a determinant factor in survival of this species in manure-amended soil (van Hoek et al., 2013), resulting in attenuated survival. Higher *rpo*S mutation rates were found in human isolates of *E. coli* O157:H7 than in the ones originating from food and from cattle, suggesting that larger variation in survival time will exist in human than in environmental isolates. No relationship was found to exist between possession of virulence genes in *E. coli* O157:H7 and survival time in manure-amended soil (Franz et al., 2011). However, from the same study, it became apparent that increased oxidizing capacities of particular metabolites such propionic acid, α-ketobutyric acid, and α-hydroxybutyric acid were important factors in survival of this pathogen in manure-amended soil (Franz et al., 2011). Many factors, thus, can influence the selection of *E. coli* strains in plant and animal production systems, and survival time of the enteric pathogens in arable soil is an important factor for eventual later plant colonization. For over almost two decades, agroproduction systems are considered to be important risk factors in the dissemination of potentially life-threatening pathogens such as *E. coli* O157:H7, whereas no information is available on the roles that pastures play in the accumulation of this type of pathogens (Jones, 1999).

The aim of this study was to demonstrate that circulation of enteric human pathogens between plants and animals is realistic, using *E. coli* as model. For that purpose, a small-scale cattle farm located in the center of Netherlands was chosen, where in the previous year *E. coli* O157:H7 was found present in feces of two rose calves. In our study, we investigated the presence of STEC in grass from the pastureland with and without grazing heifers in two successive years, by making use of cultivation-independent and dependent microbiological detection techniques. Via isolation upon enrichment, STECs can be isolated from different environments (grass shoots, rhizosphere soil, and manure), and genomic comparisons can be made between obtained isolates and *E. coli* strains from culture collections representative of different phylogenetic groups.

## Materials and Methods

### Nijkerk Farm Location

**FIGURE 1:**
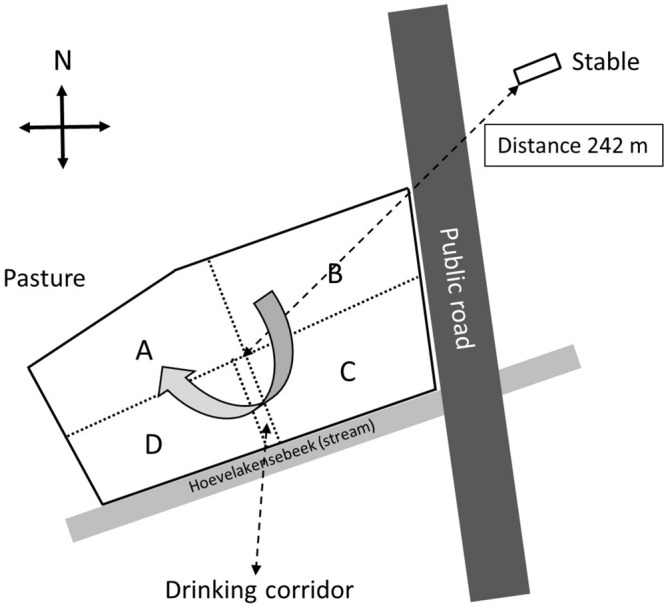
Schematic overview of the surveyed farm near Nijkerk including the stable with 50 calves and the pasture land with 15 heifers (grazing heifers were only present in 2014). The pasture was separated by electric wiring into four sectors (A through D) and a drinking corridor to allow the heifers to drink from a water reservoir near the stream. The heifers were weekly, clockwise, rotated to another sector with freshly grown grass, and at the moment of the first sampling (August 2014), the heifers were located in sector A.

A small-scale cattle farm located in the neighborhood of the village of Nijkerk, Netherlands, was selected for screening for eventual presence of *E. coli* O157:H7 and other Shiga toxin-producing *E. coli* (STEC) bacteria in pasture land and stable manure. The reason for selection of this farm was a previous finding made by the faculty of animal health sciences of the University of Utrecht, Netherlands, that *E. coli* O157:H7 was present in feces of two out of 50 rose calves during a survey over Dutch farms in April 2013. The farmer used pasture land for grazing by 15 heifers until 2014, whereas in a separate stable, 50 rose calves were housed (Figure 1). Heifers and calves never had direct contact with each other before and during the study. The distance between stable and pasture land was 242 me (in a direct line from the stable to the middle of the field), and there was no direct contact between stable and pasture land with the exception of one single yearly application of manure, via injection into soil, in March 2014 and March 2015.

### Sampling of the Nijkerk Farm

The farm was visited for sampling at three occasions in August 2014 and in April and August 2015. In 2014 and 2015, there were 50 rose calves at the age of between 6 and 7 months present in the stable. The calves were distributed over 12 compartments and per compartment samples, composed of five subsamples of 10 g of fresh manure, taken in August 2014 and stored in plastic bags (Table 1). In August 2015, 10 g of fresh manure samples were taken from two randomly selected stable compartments. The size of the grazed pasture land was 2.42 ha, and the land was separated into four sectors of about equal sizes (A through D) by electric fences (Figure 1) to allow grass plants to recover and grow for 3 weeks in the absence of grazers. In 2014, a herd of 15 heifers was present on the pasture land, whereas in 2015, the pasture land was not used for grazing anymore by heifers or any other grazers. At the moment of sampling in 2014, the heifers were located in a sector that was denoted by “A.” Based on information provided by the farmer, the presence of the heifers in the weeks before sampling was known, which was 1 week before sampling for sector D, 2 weeks before sampling for C, and 3 weeks before sampling for B. Five replicate samples were taken from each sector, and in addition, five samples were taken adjacent to separate cowpats in sector D only, resulting in a total of 25 samples in August 2014 (Table 1). The cowpats in sector D were not older than 2 weeks, and samples were taken at distances of not more than 5 cm from the cowpat where grass was still alive and green and not decolorized by ammonia emission from the cowpat. In April and August 2015, one composite sample per sector, composed of four subsamples from different sites in each sector, was taken from the pasture at both occasions. At all field sampling dates, grass cover was sampled using a soil bore (diameter of 6 cm) to a maximal depth of 8 cm. Grass shoots were separated from the roots using sterilized scissors, and roots with adhering soil were shaken by hand to remove excessive soil from the roots. Soil that remained attached to roots was considered as “rhizosphere soil.” Grass shoots and roots with rhizosphere soil were placed in plastic bags for transport to the laboratory. A corridor was created with electric fence to allow the heifers to move from their sector to a drinking place, which was adjacent to a small stream (Figure 1). The location nearby the drinking place appeared muddy because of excessive water spill and grass cover disturbance by the heifers. Because of its proximity to the drinking place, the stream was sampled for eventual presence of STECs because of the observed run-off of muddy water mixed with cow feces. Water from the stream flanking the grazed pasture land was sampled at one occasion in August 2014. At this occasion, two 50-ml water samples were taken at different locations at a distance of 10 m from each other (Table 1). All samples were kept at 4°C during transport and sample processing, and the period between sampling and sample processing never exceeded more than 4 h. Short before and during samplings in 2014 and 2015, the weather conditions were normal for the time of the year in Netherlands, and the mixed grass-clover coverage appeared normal, devoid of any yellow decolorization due to nutrient limitation or drought stress.

**TABLE 1:** Number of samples taken at the Nijkerk cattle farm location in 2014 and 2015.

**Month**	**Stable**	**Sector**	Plants*	**Water**
August 2014	12			2
		A	5	
		B	5	
		C	5	
		D	5	
		D (near dung)	5	
April 2015	0	A	1	
		B	1	
		C	1	
		D	1	
August 2015	2	A	1	
		B	1	
		C	1	
		D	1	

* Grass cover samples including roots and shoots were taken from the field and later separated in shoot and rhizosphere soil, i.e., soil attached to roots, samples.

### Sample Processing

For rhizosphere soil sample analyses, 10-g roots with adhering soil were suspended in 30 ml of 1/4 strength Ringers (Merck, Darmstadt, Germany) solution (further denoted as “Ringers solution”) in sterile 100-ml Erlenmeyer flasks with 1 g of gravel. Suspensions were shaken at 20°C for 20 min at 150 rpm. Volumes of 100 μl of the thus obtained rhizosphere soil suspensions were directly plated onto fresh CHROMagar^TM^ O157 plates (Becton Dickinson GmbH, Heidelberg, Germany) for eventual STECcolony formation. CHROMagar^TM^ O157 is a semi-selective medium for a broader group of *E. coli* strains than just *E. coli* O157:H7, however, most likely not supporting growth of all non-O157:H7 STECs present in complex microbial habitats (Fan et al., 2018). Only colony types resembling the color (mauve) and morphology of *E. coli* O157:H7 strain EDL933 (ATCC^®^ 43895^TM^) on this medium were counted and eventually selected for isolation. Mauve-stained colonies from all studied habitats were presumed to be STECs, although their identities still need to be confirmed by molecular serotyping and genomic analysis for presence of *stx*, and other STEC-related virulence genes such as *eae* and *ehx*A genes. Plates were incubated at 37°C for 16 h, including one control plate with *E. coli* O157:H7 strain EDL933 streaked to purity as reference. From the same rhizosphere soil suspensions, enrichment cultures were prepared using the ISO16654: 2001 “Horizontal method for the detection of *Escherichia coli* O157” protocol^1^. For that purpose, 1 ml of rhizosphere soil suspension was added to 9 ml of modified tryptone soya broth amended with 20 μg of Novobiocin (mTSB-N) and incubated for 18 h at 41.5°C, all in accordance with the ISO 16654 protocol. After incubation, between 2 and 10 loop-full subsamples (10 μl) of the pre-enriched cultures were streaked to purity on fresh CHROMagar^TM^ O157 plates, and plates were incubated at 37°C for 16 h after which colonies were inspected for mauve colorization. For DNA extraction, 2 ml of rhizosphere suspension from each sample were centrifuged at 7,000 × *g* for 1 min in an Eppendorf centrifuge and resulting pellets were immediately frozen and stored at −70°C. DNA was extracted from the frozen pellets using the PowerMag^®^ Soil DNA isolation kit, optimized for KingFisher^®^ application (Qiagen, Hilden, GE), according to the protocol provided by the manufacturer.

For grass shoot sample analyses, 5 g of fresh grass shoots were transferred to 12 × 14-cm size Bioreba (BIOREBA AG, Reinach, Switzerland) extraction bags containing 3 ml of Ringers solution amended with 60 μg of sodium diethyldithiocarbamate trihydrate (DIECA; Merck, Darmstadt, Germany), to prevent oxidation of the grass samples during and after homogenization. Grass shoots were homogenized by hammering for 1 min using a plastic hammer and a volume of 100 μl of filtered grass shoot homogenate per sample was used for direct plating onto CHROMagar^TM^ O157 plates, for STEC colony recovery, whereas a volume of 1 ml per sample was added to 9 ml of mTSB-N for STEC enrichment. Further incubation and processing steps for STEC colony growth were identical as described for rhizosphere soil sample analysis. For DNA extraction, 15 g of grass shoot samples were first freeze dried, resulting in dry weight samples of approximately 0.75 g, followed by grinding. Subsamples of 0.25 g of dried and ground shoots were used for DNA extraction using the same PowerMag^®^ Soil DNA isolation kit as used for rhizosphere soil DNA extraction.

For stable manure sample analyses, 10 g of stable manure per sample was suspended in 30 ml of Ringer solution, shaken at 150 rpm in 100-ml Erlenmeyer flasks at 20°C for 20 min, and 1-ml subsamples of the resulting suspensions were used for STEC enrichment according to the same procedure as described for grass shoot and rhizosphere soil sample analyses. DNA was extracted from frozen pellets (−70°C) from 2 ml of centrifuged (7,000 rpm for 1 min) manure suspensions, using the Fast DNA Stool Mini Kit (Qiagen), according to the protocol provided by the manufacturer.

For STEC enrichment from stream water samples, 1-ml volumes were added to 9 ml of mTSB-N, and mixtures were further processed as previously described for STEC enrichment from grass shoot, rhizosphere soil, and stable manure samples. Colonies, presumed to be STECs, from all enrichments from grass shoot, rhizosphere soil, stable manure, and stream water samples on CHROMagar^TM^ O157 plates were streaked to purity on fresh CHROMagar^TM^ O157 plates, and single colonies were grown in Luria Bertani [LB; peptone (Oxoid), 10 g; yeast extract (Oxoid), 5 g; sodium chloride, 5 g; water, 1 L] broth under shaking (180 rpm) at 37°C for 16 h. Thus, the obtained end-logarithmic phase cell cultures were stored frozen in LB broth with 20% glycerol at −70°C.

### DNA Extraction From Presumptive STECs and Downstream Molecular Analyses of Genomic and Environmental DNA Extracts

DNA was extracted from end-logarithmic phase cell cultures of all presumptive STECs using a “chelex DNA purification” method. For that purpose, 300 μl of cell culture was thoroughly mixed with chelex-100 resin, 50–100 mesh (BioRad) and heated to 100°C for 11 min and then cooled down to 4°C. After precipitation of the chelex resin, the cell lysate, containing between 10 and 25 ng per μl of DNA, was used for downstream analysis using PCR-nucleic acid lateral flow immunoassay (NALFIA; see below). For whole genome DNA sequencing, DNA was extracted from end-logarithmic cell cultures using the “Wizard magnetic DNA purification kit for food” (Promega), resulting in DNA yields of between 1 and 2 μg. DNA solutions (50 μl) containing between 1 and 1.5 μg of DNA were used for whole genome DNA sequencing performed by the Wageningen Plant Research Bioscience group using the Illumina MiSeq platform.

PCR-NALFIA, designed and evaluated for detection of STECs from cattle feces (Noguera et al., 2011), was applied. The following genes were PCR amplified: *E. coli* 16S rRNA (covering a broader group of species including *E. coli*, *Escherichia vulneris*, and *Shigella* species) (Huijsdens et al., 2002), intimin (*eae*), hemolysin (*ehx*A), and Shiga toxins 1 and 2 (respectively, *stx*_1_ and *stx*_2_). Positive PCR reactions were visualized by NALFIA using nitrocellulose strips containing colloidal carbon nanoparticles attached to neutravidin. Polyclonal antibodies against digoxigenin and the different fluorescent tags then immobilized at different positions specific for each fluorescent tag–primer system combination. Double-labeled amplicons were sandwiched between immobilized antibodies and the carbon–neutravidin conjugate, resulting in visualization of positive reactions by appearance of gray/black stripes at positions where amplicons were sandwiched (Noguera et al., 2011).

Draft genomes of 35 presumptive STECs were analyzed using the CLC genomics workbench 10 (Qiagen). First, phylogenetic relatedness between the 35 presumptive STECs obtained in this study with 15 *E. coli* and one *E. fergusonii* strains was determined based on DNA sequences, often cellular household genes, i.e., adenylate kinase (*adk*, 645 bp), class II fumarate hydratase (*fum*C, 1,404 bp), glycerol kinase (*glp*K, 1,509 bp), DNA gyrase subunit B (*gyr*B, 2,415 bp), 3-isopropylmalate dehydrogenase (*icd*, 1,092 bp), diaminopimelate decarboxylase (*lys*A, 1,263 bp), malate/lactate/ureidoglycolate dehydrogenase (*mdh*, 1,086 bp), methionine-tRNA ligase (*met*G, 2,034 bp), adeylosuccinate synthetase (*pur*A, 1,299 bp), and DNA recombination/repair protein (*rec*A, 1,062 bp). A maximum likelihood phylogenetic tree, with 1,000 bootstrap iterations, was constructed in MEGA 6 using concatenated and aligned loci. Reference genomes belonging to *E. coli* phylogenetic groups A (O9:H4 strain HS, K12 strain MG1655), B1 (O104:H4 strain 2011C-3493, O104:H4 strain 55989, O103:H2strain 12009, O152:H28 strain SE11, O26:H11 strain 11368, O111:H8strain 11128, O8:H9 strain IAI1), B2 (O150:H5 strain SE15, and O18:H7 strain UT189), D (O17:K52:H18 strain UMN026), E (O157:H7 Sakai strain), F (O7:H45 strain IAI39), and *E. fergusonii* ATCC35469 (as outgroup), all obtained from public databases, were used for assignment of the presumptive STECs to phylogenetic groups. Then, these presumptive STECs were *in silico* serotyped for identification of the outer membrane (O) antigen and flagellin protein (H) types using the SerotypeFinder tool from the Centre for Genomic Epidemiology (CGE) website^2^ (Joensen et al., 2015). For identification of virulence genes, six loci from the *E. coli* O157:H7 Sakai strain complete genome, i.e., *stx*_1__a_ (948 bp), *stx*_1__b_ (270 bp), *stx*_2__a_ (960 bp), *stx*_2__b_ (270 bp), *ehx*A (998 bp), and gamma intimin gene *eae* (2,805 bp) were selected and mapped against the genome sequences of the presumptive STECs. This Whole Genome Shotgun project consisting of genomes of 35 strains was deposited at the NCBI database under reference of PRJNA624229^3^.

## Results

### Grass, Manure, and Water Analysis by Application of Cultivation-Independent and -Dependent Microbial Approaches

Grassland sampling in August 2014 resulted in DNA extracts of high quality (high molecular weight DNA, low in DNA polymerase-inhibiting impurities) from 25 grass rhizosphere soil and corresponding grass shoot, and of 12 stable manure samples (Table 1). In April and August 2015, high-quality DNA extracts from four rhizosphere soil and corresponding grass shoot samples were obtained at both occasions and from two manure samples in August 2015 only. All 47 community DNA extracts were used for downstream molecular analyses in the presence of *E. coli* and virulence genes relevant for *E. coli* O157:H7 and other STECs.

Direct plating of grass shoot and rhizosphere soil samples taken in August 2014 on CHROMagar^TM^ O157 resulted in absence of any mauve-stained colonies (identical to *E. coli* O157:H7 on CHROMagar^TM^ and presumed to be STEC) on samples from grass shoots, with the exception of grass shoots near cowpats in sector D. In these samples, between 0 and 200 mauve colonies were found on plates that had received undiluted extract, resulting in presumptive STEC numbers of between log [_10_Log (*n* + 1)] 0 and 4.78 CFU (average of 3.11) per g of fresh grass near cowpats in sector D. In the rhizosphere soil, numbers of mauve colonies on plates that had received undiluted rhizosphere soil suspensions were between 0 and 96, including samples taken near cowpats, resulting in log CFU numbers of between 0 and 2.55 CFU per g of dry soil in all rhizosphere soils. Prevalence of detectable mauve colony forms in rhizosphere soil was low; five samples of 25 were found positive, of which two came from samples near cowpats. Isolation of mauve colony types (three from each positive sample) by streaking to purity on fresh CHROMagar^TM^ O157 plates never resulted in single isolated mauve colonies. Either colonies initially colorizing mauve turned blue after purification (other *E. coli* and non-*E. coli* strains different from the reference *E. coli* O157:H7 strain EDL933), or mauve colony types were overgrown by blue-stained colonies. Repeated purification steps from these mauve colony types on fresh CHROMagar^TM^ O157 again remained futile. It was, therefore, concluded that STEC quantification in grass shoot and rhizosphere soils via direct plating might give an indication on the number of presumptive STECs in these environments, but confirmation of these numbers cannot be provided because of failure in subsequent purification steps required for further identification.

Therefore, enrichments for presumptive STECs from these samples, using the ISO 16654 protocol including colony to purity streaking on CHROMagar^TM^ O157, was performed, which resulted, after purification steps, in single isolated mauve-stained colony types. A total of 85 presumptive STECs were obtained from grass shoots, rhizosphere soil, and manure over both years (Table 2). Three presumptive STECs were from rhizosphere soil sample 90 (sector B) by three independent enrichments from the same sample resulting in strains N116–N118. Four presumptive STECs were obtained from grass shoot sample 65 (sector A), and all four came from the same enrichment of the same sample, and these strains were denoted as N102–N105. In two other grass shoot samples, 70 (sector B) and 78 (sector D), respectively, six (N106–N111) and four (N112–N115) strains were obtained, all by independent enrichments. Further, two presumptive STECs were obtained from two independent stream water samples (denoted as N119 and N120), and 60 presumptive STECs were obtained from 60 independent enrichments from stable manure samples. The reason that multiple strains were isolated from the same enrichment (N102–N105) or from different enrichments of the same sample (N106–N118) was to check whether phenotypically and genotypically different variants might arise from the same enrichment or from different enrichments of the same sample. In April 2015, no presumptive STECs were obtained via enrichment from all eight rhizosphere soil and corresponding grass shoot samples. In August 2015, presumptive STECs were found in four independent enrichments from rhizosphere soil samples from sector A (N164), sector B (N165 and N166), and sector C (N163) and in two grass shoot samples from sector A (N167) and sector B (N168).

**TABLE 2:** Identities and presence of virulence genes in Shiga toxin-producing *Escherichia coli* (STECs) isolated from different ecosystems over 2 years from the Nijkerk cattle farm.

**Ecosystem,**	**Number of**	**Gene identification on the basis of PCR-nucleic acid lateral flow**						
sector and year*	**STECs (identity)**	**immunoassay (NALFIA) and confirmed by whole genome sequencing**						
		**O-type**	***stx*_1__a_**	***stx*_1__b_**	***stx*_2__a_**	***stx*_2__b_**	***ehx*A**	***eae***
RS-B-2014	3 (N116–118)	O150:H2	+	+	+	+	+	+
GS-A-2014	4 (N102–105)	O76:H7	-	-	-	-	-	+
GS-B-2014	6 (N106–111)	O150:H2	+	+	+	+	+	+
GS-D-2014	4 (N112–115)	O150:H2	+	+	+	+	+	+
SW-2014	2 (N119, 120)	O83:H42	-	-	-	-	-	-
SM-2014	60, of which 10 randomly selected:							
	5 (N7, 14, 15, 17, 20)	O150:H2	+	+	+	+	+	+
	2 (N10, 62)	O98:H21	+	+	-	-	+	+
	1 (N64)	O84:H2	+	+	-	-	+	+
	1 (N12)	O157:H7	-	-	+	+	+	+
	1(N19)	O177:H25	-	-	+	+	+	+
RS-A-2015	1 (N164)	O142:H25	-	-	-	-	-	-
RS-B-2015	1 (N165)	O89:H38	-	-	-	-	-	-
	1 (N166)	O128:H2	-	-	-	-	-	+
RS-C-2015	1 (N163)	O142:H25	-	-	-	-	-	-
GS-A-2015	1 (N167)	O128:H2	-	-	-	-	-	+
GS-B-2015	1 (N168)	O172:H28	-	-	-	-	-	-

* RS, rhizosphere soil; GS, grass shoots; SW, stream water; SM, stable manure; A through D, different field sectors; 2014, 2015, years of sampling.

### Prevalence of *E. coli* and STEC Virulence Genes in Grass, Manure, and Water Samples

**FIGURE 2:**
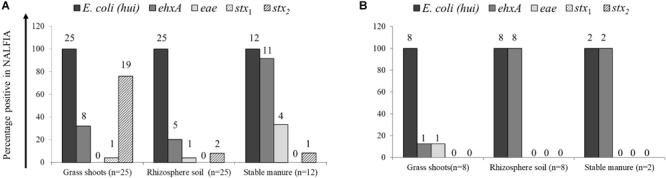
Prevalence of *Escherichia coli* 16S rRNA and virulence genes, as determined by PCR-nucleic acid lateral flow immunoassay (NALFIA), in DNA extracts from grass shoots, rhizosphere soil, and stable manure taken in August 2014 **(A)** and August 2015 **(B)** and expressed as fractions of total sample numbers from each ecosystem. Number above bars represents the absolute numbers of positive reactions per target gene for each ecosystem.

**FIGURE 3:**
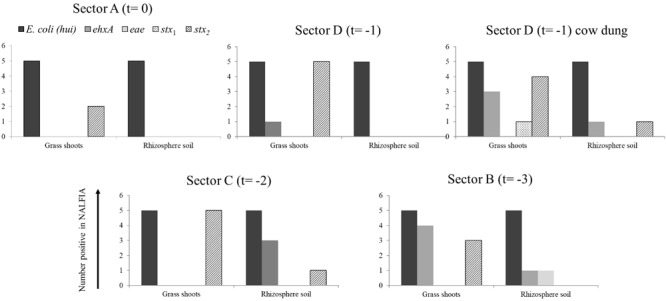
Number of positive PCR-NALFIA reactions to *E. coli* 16S rRNA and virulence genes in DNA extracts from grass shoots, rhizosphere soil, and stable manure taken in different sectors of the pasture in August 2014 (A). Numbers on vertical axis represent the number of positive NALFIA reactions for each target gene in grass shoots and rhizosphere soil DNA extracts for each sector (*n* = 5).

The presence of *E. coli* and closely related *E. vulneris* and *Shigella* species (16S rRNA gene) and STEC virulence genes *stx*_1_ and *stx*_2_ (Shiga toxins 1 and 2 genes, respectively), *eae* (intimin gene), and *ehx*A (hemolysin gene) was demonstrated using the PCR-NALFIA diagnostic platform, on community DNA samples from grass shoots, rhizosphere soil, and stable manure (Figures 2, 3). Over all samples taken in 2014 (*n* = 37), *E. coli* was found present in all grass shoot (*n* = 25) and corresponding rhizosphere soil samples and in all stable manure samples (*n* = 12) (Figure 2). The Shiga toxin *stx*_1_ gene was found in one grass shoot and in none of the rhizosphere and stable manure samples, whereas *stx*_2_ was found present in 19 grass shoot, two rhizosphere soil, and in one stable manure samples. The hemolysin gene *ehx*A was found to be present in eight grass shoot, five rhizosphere soil, and 11 stable manure samples, the intimin gene *eae* in zero grass shoot, one rhizosphere soil, and four stable manure samples. Over the 18 grass shoot, rhizosphere soil, and manure samples from April and August 2015, *E. coli* was also found present in all samples, whereas the *ehx*A gene was present in only one grass shoot sample, in all eight rhizosphere soils and in both manure samples. Further, the *eae* gene was only present in one grass shoot sample and not found in any of the other samples, whereas the *stx*_1_ and *stx*_2_ genes were not found in any of the 18 samples taken in 2015. Based on these data, it is obvious that *E. coli* was always detectably present in all community DNA samples, whereas most virulence genes and especially the *stx*_2_ gene were only present in 2014 samples, when heifers were grazing on the pasture land and absent in 2015 samples when no heifers were grazing anymore. Over all 2014 samples, the highest prevalence for *stx*_2_ was found in the grass shoots, whereas the highest prevalence for *ehx*A was found in stable manure.

Divided over the four sectors of the pasture land in 2014 (Figure 3), the *stx*_2_ gene was found present in one grass shoot sample in sector A, the sector where heifers were present at the moment of sampling. However, the *stx*_2_ gene was present in all five grass shoot samples from sectors D and C; the sectors where heifers were grazing, respectively, 1 and 2 weeks before sampling and in three samples from sector B (3 weeks before sampling). Of the other virulence genes, only the *ehx*A gene was found present in grass shoots from sector D (one sample) and B (four samples). This indicates that the *stx*_2_ gene, and occasionally the *ehx*A gene, is present in the microbial community associated with grass shoots, even in the absence of grazers for 3 weeks. In rhizosphere soil, the picture was different from that of the corresponding grass shoots, namely the *stx*_2_ gene was only found in one sample from sector C, whereas the *ehx*A and the *eae* genes were both found at one occasion in samples from sector B. Rhizosphere soil and corresponding grass shoot sampling most proximate to a cowpat in sector D also revealed consistent presence of *E. coli* in all 10 samples, and presence of the *stx*_2_ gene in four grass shoot and one rhizosphere soil sample. Shiga toxin 1 gene was only found once in a grass shoot sample and the *ehx*A gene three times in grass shoot and one time in a rhizosphere soil sample. Overall a higher prevalence of the *stx*_2_ gene was found in grass shoots in comparison with rhizosphere soil. A cultivation-based approach was applied to demonstrate the presence of *stx*_2_ genes in STECs.

Full genome analysis was, therefore, performed on DNA extracts made from all 25 presumptive STECs from grass shoots, rhizosphere soil, and stream water (over both years) and from a randomly selected set of 10 STECs from stable manure samples taken in 2014 and denoted as N7, N10, N12, N14, N15, N17, N19, N20, N62, and N64 for further downstream molecular typing.

### Identity of Presumptive STECs From the Nijkerk Farm

Identification of the 35 STECs was performed on the basis of full genome DNA sequence analysis by: (1) multilocus sequence typing of 10 *E. coli* household genes, namely, *adk*, *fum*C, *glp*K, *gyr*B, *icd*, *lys*A, *mdh*, *met*G, *pur*A, and *rec*A, (2) *in silico* serotyping using the CGE website, and (3) gene mapping for *stx*_1__a_, *stx*_1__b_, *stx*_2__a_, *stx*_2__b_, intimin gene *eae*, and hemolysin gene *ehx*A from the *E. coli* O157:H7 Sakai strain genome. The presence of the four virulence genes in draft genomes of 16 strains from grass shoots (N102, N106, N112), rhizosphere soil (N116), stream water (N119 and N120) and manure (N7, N10, N12, N14, N15, N17, N19, N20, N62, and N64) corresponded to observations made by PCR-NALFIA on DNA extracts made from these strains (Table 2).

**FIGURE 4:**
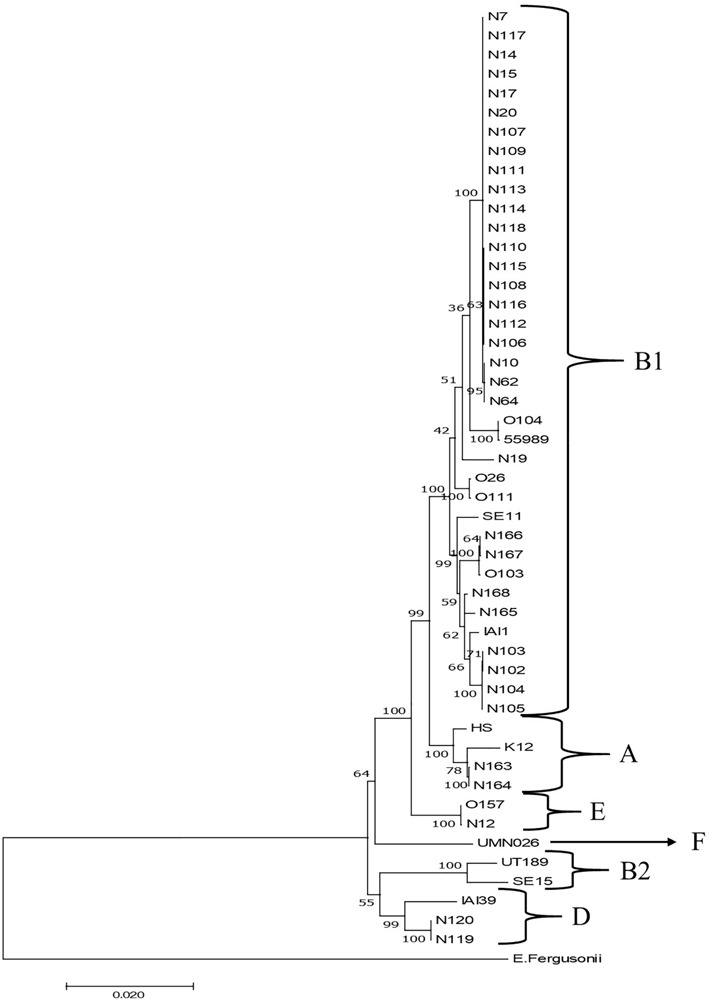
Maximum likelihood phylogenetic comparison of 35 STECs from different ecosystems on the Nijkerk cattle farm and 15 *E. coli*/*Escherichia fergusonii* strains. The tree was rooted using *E. fergusonii* (ATCC35469) as outgroup. Phylogenetic relationships between *E. coli* strains were inferred by making use of 10 concatenated household genes (*adk*, *fum*C, *glp*K, *gyr*B, *icd*, *lys*A, *mdh*, *met*G, *pur*A, and *rec*A) with 1,000 bootstrap iterations. Relevant phylogenetic groups (A, B1, B2, D, E, and F) are indicated in the tree. Reference strain abbreviations: HS, O9:H4 strain HS; K12, O16:H48 K12 strain MG1655; O104, O104:H4 strain 2011C-3493; 55989, O104:H4 strain 55989; O103, O103:H2 strain 12009; SE11, O173:H4 strain SE11; O26, O26:H11 strain 11368; O111, O111:H8 strain 11128; IAI1, O8:H9 strain IAI1; SE15, O150:H5 strain SE15; UT189, O18:H7 strain UT189; UMNO26, O17:H18 strain UMN026; O157, O157:H7 Sakai strain; IAI39, O7:H45 strain IAI39; *E. fergusonii*, *E. fergusonii* ATCC35469.

Phylogenetic clustering on the basis of multilocus DNA sequence similarities revealed one cluster of 21 strains that closely resembled each other on the basis of MLST (Figure 4). These 21 strains showed phylogenetically closest relationship with two *E. coli* O104:H4 strains and more distinct relationships with five other *E. coli* strains, namely, O103:H2, SE11, O26:H11, O111:H8, and IAI1, all from phylogenetic group B1. Ten of these strains originated from grass shoots, eight from stable manure samples, and three from rhizosphere soil, all from 2014. Of the 10 grass shoot strains, six were from sector B and four from sector D. Altogether, these 21 strains originated from 11 independent samples. The fact that closely resembling strains originated both from the stable manure (eight) and from pasture field (three) samples indicates that interactions between stable and pasture land must have taken place in spite of the fact that both were distantly located from each other. These 21 strains also resembled each other on the basis of presence of virulence genes, i.e., all six tested virulence genes were present in the genomes of 18 strains, whereas in three strains, both *stx*_2_ genes were shown to be absent (Table 2). The 18 strains containing all virulence genes, including *stx*_2_ genes were identified to belong to the same O150:H2 *E. coli* serotype, whereas the three other strains (all originating from manure) were typed to belong to O98:H21 (two) and O84:H2 (one) *E. coli* serotypes. Further, only one strain, N12, only containing *stx*_2_ and no *stx*_1_ genes, from stable manure, was serotyped as *E. coli* O157:H7 (phylogenetic group E), and this was a remarkable fact because it was anticipated on forehand that the *E. coli* O157:H7 serotype would prevail among the manure isolates. One other strain from manure was shown to possess *stx*_2_, and no *stx*_1_ genes in its genome and this strain was identified to belong to *E. coli* serotype O177:H25 (N19). Altogether, 23 strains, of which 22 belonged to phylogenetic group B1 and one to E, carried one or more *stx* genes in their genomes, and therefore, these 23 strains were confirmed to be STEC positive. Twelve strains did not possess any *stx* gene in their genomes, and thus, these strains are further denoted as “non-Shiga toxin-producing *E. coli* strains,” i.e., non-STECs. These non-STECs did not cluster together in the phylogenetic tree, and most (8) belonged to phylogenetic group B1, two to A and two to D. Two strains, denoted as N166 and N167 and containing the intimin *eae* gene, were closely related to *E. coli* O103:H2. Both strains originated, respectively, from grass shoots and rhizosphere soil taken in August 2015 and were identified to belong to *E. coli* serotype O128:H2. All other 10 non-STECs were shown to belong to different serogroups (O142:H25, O76:H7, O83:H42, O89:H38, O172:H28) and must be considered as “commensal” *E. coli* isolates. Of these 10, two, denoted as N163 and N164 (O142:H25), and showing closest relationship with *E. coli* K12, were from rhizosphere soil sampled in August 2015. Four strains, denoted as N102–N104 (O76:H7), and showing closest relationship with *E. coli* strain IAI1, were from grass shoots sampled in 2014, and their resemblance was expected on forehand because these four strains originated from the same enrichment culture. Two non-STECs, originating from stream water adjacent to the pasture land (N119 and N120 and serotyped as O83:H42), showed closest relationship with *E. coli* strain IAI39 and most likely belong to phylogenetic group D. These two strains were most distinct from the other 33 strains, indicative of the fact that stream water may carry other *E. coli* strains than present in pasture land and stable manure. Two other non-STECs, N165 (O89:H38) from rhizosphere soil from August 2015 and N168 (O172:H28) from grass shoots from August 2015, showed close relationship with each other and with strains N102–N104, and belonged to phylogenetic group B1.

In summary, it is obvious that most of the STEC and non-STEC (commensal *E. coli*) strains obtained from the pasture land in 2014 and 2015 belonged to phylogenetic group B1. The majority of these 2014 strains were STECs and closely resembling strains isolated from stable manure. The non-STECs from this phylogenetic group from 2014 (strains N102–N104) were closely related with four strains from pasture land from 2015.

## Discussion

The presence of STECs and *stx*_2_ genes on grass shoots and in rhizosphere soil at the first sampling, when heifers were grazing on the pastureland, but not in the second and third samplings, in the absence of grazing animals, indicate a positive relationship between grazers and STECs/*stx*_2_-phages in pastureland. This is a main conclusion that can be drawn from this study. The fact that the farmer would withhold his grazing animals from the pastureland in the second year was not anticipated on forehand in the design of our study. His decision was fortunate to us because now we were able to investigate the same pastureland in the absence of grazing animals during the summertime of the succeeding year. Stable manure was applied to the pastureland in the same way in both years, which indicates that manure application, itself, may not be enough to cause field contamination with STECs or *stx*_2_-phages. Potential risks, indeed, exist with respect to persistence of human pathogens in agricultural environments as already stated before in Jones (1999), but now, with the implication of grazing animals on pasture land, that can lead to contamination of grass plants with non-typical STECs.

It has been shown before that *E. coli* O157:H7 and *S. enterica* can survive for over a month in bulk and rhizosphere soil and that both species can percolate to deeper soil layers, depending on manure type and type of manure application to soil (Semenov et al., 2009). It is, thus, realistic to presume that *E. coli* strains present in stable manure were able to survive long enough in deeper soil layers upon manure injection into soil to make contact with growing grass roots. The *E. coli* strains could establish themselves in the rhizosphere soil by consumption of root-released nutrients. Further spread to above-soil parts of the grass plants might be possible either actively via internal plant colonization, or passively, e.g., at rainfall via splashing of water droplets with contaminated soil to the above-soil parts of the grass plants. However, it was active grazing by the heifers instead of stable manure application to soil that was the main driving factor behind STEC contamination of the grass plants. Grazing animals sometimes pull out grass from the soil that, itself, may lead to smearing of soil with contaminating agents over the pasture. However, excrements from grazers may be held responsible for most of the contamination of the pasture. *E. coli* was shown to persist in decomposing cow pats and neighboring soil for 6 months and over in Muirhead (2010). In our study, the presence of *stx*_2_ and other virulence genes near and on grass plants in the neighborhood of heifer pats did not seriously deviate from the other investigated locations in the pasture. This would indicate that contaminating agents may be spread from the pats over the pasture, and most likely smearing occurred via the hooves of the grazers when walking over the land.

The near clonal group of 21 STECs, belonging to phylogenetic group B1 and typed as *E. coli* O150:H2, O98:H21, and O84:H2 and carrying three (O98:H21 and O84:H2) or four (O150:H2) of the virulence genes screened for, was found in two physically separated locations in this study, namely, in stable manure and near and on grass plants at the first sampling event. The fact that stable manure applications into the field in early springtime of both years were the only clear links between stable and pasture may indicate that a single origin of this particular group of *E. coli* strains was present. The fact that *E. coli* O150:H2 was only found at the first sampling may be a consequence of grazers on the pasture. A possible explanation can be that stable manure injection into the soil led to field contaminations with this *E. coli* line. When the heifers were present, *E. coli* O150:H2-contaminated grass with roots were ingested by the grazers. Uptake of *E. coli* O150:H2 resulted in outgrowth of the *E. coli* line in the intestinal track system of the animals, and excretion of larger quantities of *E. coli* O150:H2 were returned to the pasture via their feces, from where *E. coli* O150:H2 was further smeared over the field. Without grazers, no outgrowth, and further smearing of *E. coli* O150:H2 occurred, explaining the absence, or below the limits of detection, of this particular bacterial line in the second and third sampling events in 2015. This explanation is in favor with our hypothesis that human pathogens likely circulate between plants as feed and grazing herbivores such as (young) cattle. The plant must be considered as an alternative environment for this particular group of potential human pathogens waiting for the opportunity to colonize intestinal track systems of other grazing animals and from an ecological perspective creating the opportunity to disseminate over other herds of grazing animals feeding from the same land.

*Escherichia coli* O150:H2 is not often found as contaminating agent in foods and environments, although it has been described as an emerging serogroup for over several years (Monaghan et al., 2012; Onyeka et al., 2020). The group of 21 closely resembling STECs from our study showed closest relationship with strains belonging to phylogroup B1, the group to which *E. coli* O104:H4 strains belong to. *Escherichia coli* phylogenetic group B1 strains are known to be environmental strains, commonly occurring in different household animal species (Carlos et al., 2010) and freshwater beaches (Walk et al., 2007), whereas *E. coli* O150:H2 strains are commonly associated with beef products (Monaghan et al., 2012). The *E. coli* O150:H2 strains obtained from our study are not related to *E. coli* O150:H5, which belong to phylogroup B2; a group consisting of extra-intestinal pathogenic and uropathogenic *E. coli* strains, including the commensal strain SE15 and the multidrug-resistant sequence type ST131 (Toh et al., 2010; Petty et al., 2014). Three other strains from the same group belonged to serogroups O98:H21 and O84:H2, and all three strains carried *stx*_1_, *eae*, and *ehx*A, but no *stx*_2_ genes, and all three were derived from manure. Although closely related with the other 18 O150:H2 strains, these three strains were different on the basis of their sero- and virulotypes. Strains belonging to O98 and O84:H2 serotypes were found before in, respectively, diseased monkeys in China (Qi et al., 2017) and in humans, cattle, and sheep in New Zealand (Cookson et al., 2006). That STEC virulence genes are located on genomes of serotypes different from the most commonly observed ones can be an indication for the fact that new STEC types still arise (Hao et al., 2012). For example, it was suggested that adaptation to the plant environment could lead to more virulent *E. coli* O157:H7 types (Kulasekara et al., 2009).

Besides the closely resembling STECs found in our study belonging to serogroups O150:H2, O98:H21, O84:H2, O177:H25, and O157:H7, other less virulent *E. coli* types were found at the first (O76:H7; four strains) and third sampling events (O128:H2; two strains), respectively, in 2014 and 2015. *E. coli* O76 and O128 strains carrying *stx* genes were isolated before from humans in, respectively, Spain (Sánchez et al., 2014) and Germany (Beutin et al., 2004). In our study, strains of both serotypes did not carry *stx* and *ehx*A genes, but contained *eae* genes. The fact that these strains were found over 2 years in different habitats (grass shoots and rhizosphere soil) may imply that mild virulent *E. coli* strains are continuously present in pasture, also in the absence of grazers. Further, six *E. coli* strains with different serotypes were detected in water samples (2014) and in grass shoot and rhizosphere soil samples from 2015, and all strains did not carry any of the tested virulence genes. This indicates that a second, more diverse, group of non-virulent and commensal *E. coli* strains exist in pasture land and adjacent stream water. *Escherichia coli* has been found before in plants (Whitman et al., 2005; NandaKafle et al., 2017), and niche partition among different *E. coli* strains was observed in pasture land (NandaKafle et al., 2017). In the last study, it was proposed that certain *E. coli* types could survive winter conditions, including freezing temperatures. It is possible that these commensal *E. coli* types from our study may be representatives of *E. coli* populations that naturally reside in or on grass plants or on/in other plants (clover) that grow in pasture. Upon manure application, the more threatening *E. coli* types, among which the STECs serotyped as O150:H2, were introduced into pasture. In the absence of grazing animals in 2015, no augmentation of these STECs occurred, and only the naturally residing *E. coli* strains remained present in pasture in 2015. This would be the best explanatory model on the presence of the different *E. coli* strains in the Nijkerk farm. The fact that no STECs were found in the stream near the drinking place indicates that STECs from feces at least not vastly contaminated the water of the stream. This observation corresponds to a previous observation made in surface water bodies nearby agricultural fields in Netherlands where *E. coli* O157 was detected although without carriage of Shiga toxin production genes (Heijnen and Medema, 2006).

The presence of STECs in pastureland was confirmed by molecular analysis using PCR-NALFIA, although by following this approach, it was not possible to make a physical link between *E. coli* and the detected *stx*_2_ gene. It was obvious that *stx*_2_ genes were more prevalent in pasture in 2014 than all of the other virulence genes screened for, whereas derived *E. coli* O150:H2 strains possessed all virulence genes. The observed inconsistence in “stoichiometry” between the screened virulence genes was obvious in grass shoot and rhizosphere soil DNA extracts, and this may be related to the relatively higher selectivity of the CHROMagar ^TM^ O157 medium for serotype O157:H7 over non-O157 STECs (Fan et al., 2018), but also may be related to the fact that phage DNA containing *stx*_2_ genes were co-extracted with bacterial DNA from soil and plant samples. This corresponds to the observation made by Grau-Leal et al. (2015), where numbers of *stx*_2_ bacteriophages were found to be higher than *stx*_1_ bacteriophages in many different extraintestinal environments, including soils.

Both cultivation-dependent and independent approaches did not allow us to make any quantification of STECs or *stx*_2_ carrying microorganisms in these environments. However, based on direct CFU counts from mauve-stained colonies from the grass shoots nearby pats and in rhizosphere soil, the order of magnitude may lay between log 2 and log 3 per gram of soil or gram of fresh plant weight. These numbers occasionally may be too low for molecular detection in environmental DNA extracts, but possibly still high enough for cultivation by enrichment. Considering the fact that further purification attempts of the mauve-stained colonies on CHROMagar^TM^ O157 plates remained futile, it may be that *stx*_2_ genes were present in other STECs that were not able to grow on CHROMagar^TM^ O157 medium, or that STEC cells directly recovered from grass shoot and rhizosphere soil were seriously stressed and that a pre-enrichment step was needed to overcome cellular damage (Hepburn et al., 2002; Ishii et al., 2006).

In conclusion, this study makes clear that the combination of plants and grazing animals is the most important driver behind the establishment and augmentation of a potentially dangerous non-typical STEC population in pasture land. The circulation of human pathogens between plants as feed for grazing animals in the pasture land would be indicative for the fact that circulations of human pathogens exist at larger and more complex scale levels between plant (as feed) and animal production systems. Possibly, these circulations remained unnoticed in studies that were only focused on typical STECs, such as O157:H7. This is an important message especially with respect to safe production of healthy foods such as fresh produce (Guttierrez-Rodriguez and Adhikari, 2018). Circulation of human pathogens between different plant and animal production systems is a topic that would require more attention in safe food production programs.

## Data Availability Statement

The datasets generated for this study are available on request to the corresponding author.

## Ethics Statement

A microbiological survey was carried out on a private property. For that purpose, permission was granted by the owner of the farm. The farmer fully collaborated in the research performed on his property, but indicated to remain anonymous in scientific communications.

## Author Contributions

LO, AA, HR, and PW conceived the project and designed the experiments together with JW and PZ. PW, PZ, and LO were the main responsible persons for field surveying and microbiological analyses. JW was responsible for PCR-NALFIA analysis and data analysis. All authors contributed to the article and approved the submitted version.

## Conflict of Interest

The authors declare that the research was conducted in the absence of any commercial or financial relationships that could be construed as a potential conflict of interest.

## Footnotes

1 https://www.iso.org/standard/29821.html

2 http://www.genomicepidemiology.org/

3 https://www.ncbi.nlm.nih.gov/Traces/study/?acc=PRJNA624229
